# Blood Lead Levels among Non-Occupationally Exposed Pregnant Women in Southern Thailand

**DOI:** 10.3390/toxics10100599

**Published:** 2022-10-11

**Authors:** Donrawee Waeyeng, Tanaporn Khamphaya, Phisit Pouyfung, Udomratana Vattanasit, Supabhorn Yimthiang

**Affiliations:** 1 Environmental, Safety Technology and Health Program, School of Public Health, Walailak University, Thaiburi, Thasala, Nakhon Si Thammarat 80160, Thailand; 2 Occupational Health and Safety Department, School of Public Health, Walailak University, Thaiburi, Thasala, Nakhon Si Thammarat 80160, Thailand; 3 Environmental Health and Technology Department, School of Public Health, Walailak University, Thaiburi, Thasala, Nakhon Si Thammarat 80160, Thailand

**Keywords:** blood lead levels, lead exposure, pregnant women

## Abstract

Lead (Pb) is a heavy metal that is toxic to humans, especially children and pregnant women. In Thailand, guidelines exist to minimize lead exposure in pregnant women working in lead-related occupations. However, no guidelines exist for pregnant women who are not employed in these economic sectors. This cross-sectional study aimed to examine blood lead levels (BLLs) and related risk factors among 80 non-occupationally exposed pregnant women from the general population living in Nakhon Si Thammarat province, Southern Thailand. BLLs were determined by graphite furnace atomic absorption spectrophotometry. A validated questionnaire was adopted to interview participants which included demographic, consumer goods, supplement intake, and health factors. The mean BLL was 4.68 ± 1.55 µg/dL (95% CI 4.33–5.02) and 42.50% had BLLs ≥ 5 µg/dL. Higher education was the only demographic factor associated with BLLs ≥ 5 µg/dL (aOR 0.16, 95% CI 0.03–0.80, *p* = 0.027). Systolic blood pressure was also associated with BLLs ≥ 5 µg/dL (aOR 5.00, 95% CI 1.23–17.16, *p* = 0.023). However, consumer goods and supplement intake were not associated with BLLs. Our results indicate that pregnant women from the general population who were not in the risk exposure group had lead in their bodies. Except for education, demographics were not associated with pregnant women with BLLs. However, with health factors, even low BLLs had a small effect on systolic blood pressure. These data suggest a need for promoting health education and health interventions to prevent the dangers of lead exposure, especially for pregnant women and children.

## 1. Introduction

Lead (Pb) is a heavy metal that has detrimental and irreversible effects on humans, especially children and pregnant women [1,2]. Many countries have used leaded gasoline for the higher octane. However, its use has been banned in many countries, including Thailand, where it has been prohibited since 1996. Additionally, lead is nondegradable, which causes its accumulation at different levels of the food chain [3]. Lead is also used as a component in a range of products, such as paint, cosmetics, and pesticides [4,5]. Moreover, in lead-contaminated areas, lead can be absorbed and accumulated in different parts of plants, which means that consumers have a risk of exposure from plant sources [6]. This risk of exposure to lead can affect not only those who work with lead, but the wider population as well. Lead can enter the body through inhalation, ingestion, and skin absorption, and in the case of fetuses during pregnancy, the lead contained in the mothers’ bones may be released into the blood and pass through the placenta, exposing the fetus [7]. Currently, the gold standard method for detecting lead in the body is blood testing, but there is currently no safe blood lead level (BLL) threshold [8]. Recent studies have shown that even low BLLs in pregnant women have been linked to an increased risk of preeclampsia, high blood pressure, and anemia [2,9], as well as a negative influence on a child’s physical growth and brain functioning [10,11].

BLLs in pregnant women is a crucial public health concern in many countries [8,12]. Blood lead concentrations vary from country to country depending on factors or the likelihood of exposure to lead, for example the average pregnant woman’s BLL in Poland was 1.10 ± 0.20 µg/dL [13], 3.67 ± 1.47 µg/dL in the UK [12] and more than 5.00 µg/dL in China [14]. Many factors influence these figures, including personal health, occupation, and consumption of supplements [12,14,15]. In Thailand, there are no current data on BLLs in pregnant women, with the last study taking place in 1993, which found that the mean BLL in pregnant women was 6.20 ± 2.00 µg/dL [16]. In addition, there were studies conducted on children living near lead sources (BLL range 0.03–26.40 µg/dL) [17] and on people working with lead (range 4.59–39.33 µg/dL) [18], which were both conducted in southern Thailand.

Thailand has guidelines for the prevention of lead exposure in non-occupationally exposed pregnant women engaged in lead-related occupations [19]. However, guidelines to protect pregnant women from danger are still lacking. Previous studies have indicated the presence of lead in the blood of pregnant women, and that even low levels can affect their health and that of their children [2,9,10,14,20]. The objectives of this study were to evaluate blood lead concentration and examine the association between BLLs and demographic factors, consumer goods factors, supplement intake factors, and pregnant women’s health. This will serve as a roadmap for future lead-risk surveillance and prevention efforts.

## 2. Materials and Methods

### 2.1. Study Population

This cross-sectional study was conducted among 80 pregnant women who received antenatal care at district health promotion hospitals in Mueang and Thasala districts, Nakhon Si Thammarat province, from June to December 2021. The pregnant women enrolled in the study must have lived in Mueang or Thasala district for at least one month and must not have had an occupation that was considered at risk of lead exposure, as indicated by the Department of Disease Control, Thailand [19], including (1) lead mining, (2) battery making, (3) metal welding, metal cutting, (4) metal polishing work, (5) painting or spray painting, (6) steel work, (7) electronic and computer semiconductor factory, (8) pesticide manufacturing and packing industry, (9) ceramic factory, (10) metal jewelry factory, (11) car garage or shipyard, (12) paint industry, (13) factory manufacturing pipes, sheet metal or metal plating, (14) printer foundry, (15) ammunition factory, and (16) other occupations in which workers are exposed to lead. The number of participants was calculated according to a study by Mondal et al. (2016) [21].

N = ([Zα + Zβ]/C)^2^ + 3 (1)

where N = number of subjects required, Zα = standard deviation for α = 1.96, and Zβ = standard deviation for β = 1.64.

C = 0.5 × ln ([1 + r]/[1 − r])(2)

where r = the expected correlation coefficient = 0.39 [9].

Thus, we obtained a sample size of 80 pregnant women.

### 2.2. Data Collection

Data were collected using structured questionnaires which included demographic factors, consumer goods factors, supplement intake factors, and items related to the health of pregnant women. The interviews were conducted on the same day that the blood samples were collected.

### 2.3. Questionnaire Validation

The questions in the interview questionnaires were examined for validity and inter-rater reliability using the Item Content Validity Index (I-CVI), Scale Content Validity Index Universal Agreement (S-CVI/UA), Scale Content Validity Index Average (S-CVI/Ave), and Kappa (K) [22] by five experts in Thailand with expertise in lead research. The results for validity and inter-rater reliability were equal to one for every variable.

### 2.4. Measurement of Blood Lead

Blood samples from pregnant women were collected by nurses at each district health-promotion hospital. A syringe and needle with an anticoagulant, ethylene diamine tetra-acetic acid, were used to collect approximately 3 mL of venous blood from each pregnant woman. Blood samples were stored at −20 °C in a sealed compartment to avoid contamination during storage and transport, and sent to the Bangkok RIA Laboratory in Thailand. Sample preparation following a method described by [23]. Blood lead concentration was determined by graphite furnace atomic absorption spectrometry Agilent^TM^ 240Z AA (Agilent Technologies, Inc, Santa Clara, CA, USA). The detection limit was 1.0 µg/dL.

### 2.5. Assessment of Pregnant Women’s Biological Indicators

Biological indicators of pregnant women, including blood pressure and hemoglobin levels, were assessed as part of a routine laboratory check-up for pregnant women by health professionals at district health promotion hospitals. The blood pressure was measured using an automatic sphygmomanometer. In this study, systolic blood pressure was defined as ≥120 mmHg, and diastolic blood pressure was defined as ≥80 mmHg. Hemoglobin was measured using the sodium lauryl sulphate (SLS) method [24], with abnormal values defined as <11 g/dL.

### 2.6. Data Analysis Method

Data were analyzed using SPSS software version 28 (SPSS Inc., Chicago, IL, USA). The comparison of the mean difference of BLLs for each variable was analyzed using an independent *t*-test (two groups) or one-way ANOVA (more than two groups) with a 95% confidence interval (CI). The relationship between BLLs and pregnant women’s health was analyzed using Pearson’s correlation coefficient. Predictors of high BLLs were analyzed using logistic regression analysis.

## 3. Results

### 3.1. Pregnant Women Demographic Characteristics and BLLs

The mean BLL of 80 pregnant women was 4.68 ± 1.55 µg/dL (95% CI 4.33–5.02) and 42.50% had BLLs ≥ 5 µg/dL. The average age of the pregnant women was 27 years, with ages ranging from 18 to 42 years, with 35.00% in their first trimester, and 58.75% being multiparous. A total of 42.50% had a lower secondary level education and 52.50% were unemployed, most of whom stopped working when their gestational age reached the second trimester. The main occupation of those working during pregnancy was trading. Their husband’s occupation, such as a car mechanic, carried a risk of lead exposure of 23.75%. The median household income was 12,000 THB/month (359.17 USD) and 71.25% lived with a husband or father who smoked. We conducted tests to determine differences in BLLs in demographic characteristic factors and found that BLLs likely varied significantly with educational level (*p* = 0.049) (Table 1).

### 3.2. Consumer Goods and Supplement Intake of Pregnant Women and BLLs

There was no significant mean difference between the consumer goods factors, including coffee consumption, milk consumption, seafood consumption, eyeliner use, lipstick use, brush-on use, nail varnish use, and household insecticide use (Table 2). Similarly, there was no significant mean difference between the supplement intake factors, including iron, calcium, and vitamin C (Table 3).

### 3.3. Pregnant Women’s Health and BLLs

BLLs had a weak negative correlation with gestational age (r = −0.251, *p* = 0.024) and a weak positive correlation with systolic blood pressure (r = 0.271, *p* = 0.015). In other words, BLLs decreased when gestational age increased and BLLs increased as systolic blood pressure increased (Table 4).

### 3.4. Predictors of BLLs ≥ 5 µg/dL

The crude odds ratio (cOR) and adjusted odds ratio (aOR) of education levels and pregnant women’s health outcomes were also calculated. After adjustments, higher education was associated with an 84% reduction in the risk of having high BLLs, and pregnant women with systolic blood pressure ≥ 120 mmHg were associated with having BLLs five times higher than those with systolic blood pressure < 120 mmHg (Table 5).

## 4. Discussion

Pregnant women exposed to lead, even at low levels, face an increased risk of adverse perinatal outcomes and negative effects on their children’s development. In this study, we examined BLLs and health outcomes in pregnant women who did not work in occupations with a risk of lead exposure in Nakhon Si Thammarat province, Thailand. The mean BLL was 4.68 ± 1.55 µg/dL, lower than the maximum recommended BLL in pregnant women (5 µg/dL) [25] and the average reported in China (the mean BLL in three trimesters was 5.98 ± 2.43, 5.54 ± 2.01, and 5.59 ± 1.97 µg/dL, respectively) [14] but higher than that of the United Kingdom (UK) (3.67 ± 1.47 µg/dL) [12] and comparable to that of Bangladesh (4.7 ± 3.60 µg/dL) [15]. Differences in BLLs in pregnant women are caused by a variety of factors. For example, in the UK, previous studies found that the factors related to BLLs were cigarette smoking, alcohol and coffee drinking, and heating the home with a coal fire. Additionally, iron and calcium intake were protective factors [12]. In Bangladesh, the factors affecting BLLs were the consumption of canned foods, use of agrochemicals, and rice grinding [15], while in China, occupation, nutritional supplement intake, and the length of time since last having the home painted were associated with BLLs [14]. In developed and very high Human Development Index (HDI) countries, including the UK, USA, and Canada, measures and programs implemented to manage the risks of lead exposure and reduce lead contamination in the environment have helped reduce lead exposure to the body [26,27,28,29]. A study by Hwang [30] corroborates this, discovering that BLLs in children in very high HDI countries were lower than countries with lower HDI levels, which explains the variation in BLLs in pregnant women across different countries. Previous studies in Thailand, primarily focused on worker and children’s groups, showed that the factors related to BLLs included occupation, environment, and living near factory plants or fishing communities [17,31,32,33,34]; however, there were no data concerning factors related to BLLs in pregnant women. In this study, we examined demographic factors, consumer goods factors, and supplement intake factors. To our knowledge, this was the first study in Thailand that examined BLLs among non-occupationally exposed pregnant women, and its related factors.

Education was found to be a demographic factor associated with BLLs. Education is one of the most important factors in health and wellness behaviors; generally, people with higher education tend to be healthier and more likely to engage in healthy behaviors [35]. Education level is also used as an indirect measure of social and economic class; that is, the majority of people with good education also have a good social and economic class [36]. This is related to environmental and hygiene factors [13]. Other demographic factors in our study that were not related to BLLs included pregnant women’s age, parity, pre-pregnancy BMI, occupation, husband’s occupational risk of lead exposure, household income, and duration of current stay. As the majority of pregnant women in our study did not work in lead-related occupations, BLLs were low, and low BLLs may be unable to demonstrate the association between the aforementioned factors. Our findings are consistent with those of previous studies [14,20,37]. Demographics were not the main related factor in pregnant women with low BLLs, with the exception of education level.

Our study indicated the presence of lead in the bodies of pregnant women. However, there was no significant association between BLLs and consumer goods factors that may be the source of lead exposure. In terms of cosmetic use, most pregnant women in our study did not use cosmetics except lipstick and bought lipstick from department stores or convenience stores, indicating that most lipstick sold in these locations was manufactured according to high production and quality control standards compared to those sold at market stalls, which have been found to be contaminated with lead [5]. However, since these products have not been tested in this study, we cannot be completely certain of this.

In the case of the supplement intake, previous studies have reported that consuming iron, calcium, and vitamin C has been shown to reduce lead absorption [38,39,40]. However, our study did not find a relationship between supplement intake and BLLs, possibly because almost all participants had antenatal care at the hospital, and were receiving supplements in accordance with the standard practice of pregnant women’s care in Nakhon Si Thammarat province, following the WHO recommendations on antenatal care [41]. Pregnant women receive triferdine 150 mg/day and calcium (CaCO_3_) 1200–1250 mg/day throughout the three trimesters, and receive ferrous fumarate 200 mg/day if they exhibit iron deficiency anemia. The consumption of fruits and vegetables is promoted for obtaining vitamin C.

In terms of gestational age, a study by Hertz-Picciotto [42] described the patterns of blood lead concentration during pregnancy as a U-shaped curve, since lead stored in the mother’s bones is released into the blood through calcium metabolism. Calcium metabolism during pregnancy consists of two important phases: (1) increasing maternal blood volume during early pregnancy to maintain circulating levels of calcium, with total calcium in the blood increasing primarily through increased intestinal calcium absorption and reduced renal calcium excretion; (2) during the third trimester, fetal bone formation becomes an important factor in maternal calcium metabolism for transferring calcium to the fetus [43,44]. Thus, the first and third trimesters have high blood lead concentration. The present study found that BLLs had a weak positive correlation with gestational age, in contrast with Hertz-Picciotto’s [42] study. This can be explained by the difference in lead exposure factors, such as pregnant women’s lifestyle, supplement intake, sampling period of BLLs, and research study design, with the current study being cross-sectional. Nevertheless, the results of our study highlight a trend of lead release during the first trimester of pregnancy consistent with a study by Liu [14], who found that BLLs of pregnant women in the first trimester were higher than that of subsequent trimesters, which may affect the development of the fetus’s nervous system to a greater extent than during other trimesters.

Concerning pregnant women’s health outcomes, our study found the systolic blood pressure associated with high BLLs ≥ 5 µg/dL to be consistent with Gambelunghe’s study [45]. Previous studies have described the mechanism of lead exposure, which causes high blood pressure and oxidative stress, impairs nitric oxide (NO) bioavailability, obstructs endothelial repair, inhibits angiogenesis, and reduces endothelial cell growth [46,47,48]. However, our study did not find a correlation between BLLs and diastolic blood pressure, which is dependent on several factors, namely the measurement method used, the ability of the measurer, and other variables under which the measurement is taken [49]. Furthermore, hemoglobin was not related to BLLs due to the pregnant women’s low BLLs, a result consistent with that of La-Llave-León [50]. Most studies have found that anemia caused by lead appears at BLLs > 10 µg/dL [51,52].

The situation regarding lead exposure in pregnant women in Nakhon Si Thammarat province, Thailand is one that raises important concerns. Although pregnant women do not work in occupations that involve lead, studies have shown that pregnant women may still have BLLs greater than 5 µg/dL. To prevent lead exposure among pregnant women, we suggest that the Public Health and Education Departments should provide information on toxic substances that are harmful to the mother and fetus at the school level. In addition, pregnant women with lower education levels, a systolic blood pressure of ≥120 mmHg, and a previous job that involved lead should be thought of as being at risk of lead exposure. These could be important factors when making guidelines for pregnant women who are not exposed to lead at work. Moreover, we encourage further investigation into lead contamination in the environment, such as soil, water, air, consumer products, and cosmetics, as our results, through the presence of BLLs in pregnant women, suggest that exposure comes from the environment. Previous occupation should also be considered because lead stored in the mother’s bones from prior exposure is released into the blood during pregnancy through calcium metabolism. Nevertheless, the present study has the following limitations: (1) recall bias; the consumer goods and supplement intake in the past month were followed according to the timeframe of lead half-life; (2) the living with a smoker factor in this study did not cover exposure at the workplace; (3) the pregnant women’s occupation factor did not cover previous occupations; (4) the findings of this study may not be applicable to a group of pregnant women who receive special antenatal care at a clinic or private hospital, as demographic factors may differ.

## 5. Conclusions

Lead was detected in the blood of pregnant women who did not work in occupations where they might be exposed to lead. Having a high level of education was the most important demographic factor for low BLLs. Only systolic blood pressure was positively associated with higher BLLs. There was no association between consumer goods or supplement intake and BLLs. These findings demonstrate the necessity for health education and health interventions to protect individuals from the dangers of lead, as well as for continuous monitoring to determine what action should be taken into account.

## Figures and Tables

**Table 1:** Demographic characteristics and mean BLLs of pregnant women (*n* = 80).

Characteristics	*n* (%)	BLLs (µg/dL)		*p*
		Mean ± SD	95% CI	
Mean BLL	80 (100.00)	4.68 ± 1.55	4.33–5.02	-
Age (year)				
≤25	40 (50.00)	4.77 ± 1.64	4.25–5.30	0.567
>25	40 (50.00)	4.57 ± 1.46	4.10–5.04	
Trimester				
First trimester	28 (35.00)	5.07 ± 1.54	4.48–5.67	0.111 ^a^
Second trimester	26 (32.50)	4.73 ± 1.84	3.99–5.48	
Third trimester	26 (32.50)	4.19 ± 1.10	3.75–4.64	
Parity				
Primiparous	33 (41.25)	4.75 ± 1.39	4.26–5.25	0.692
Multiparous	47 (58.75)	4.61 ± 1.66	4.13–5.10	
Education				
Primary (7–12 years)	17 (21.25)	4.49 ± 1.67	4.08–5.80	0.049 ^a^*
Lower-secondary (13–15 years)	34 (42.50)	4.76 ± 1.71	4.14–5.38	
Upper-secondary (16–18 years)	16 (20.00)	5.06 ± 1.10	4.53–5.59	
Higher education (>18 years)	13 (16.25)	3.61 ± 0.78	3.15–4.08	
Pregnant women’s occupation				
Unemployed	42 (52.50)	4.74 ± 1.56	4.25–5.22	0.704
Employed	38 (47.50)	4.60 ± 1.55	4.09–5.11	
Risk of lead exposure through husband’s occupation				
No	61 (76.25)	4.67 ± 1.62	4.26–5.09	0.977
Yes	19 (23.75)	4.68 ± 1.33	4.04–5.33	
Household income (THB/month)				
<9000	31 (38.75)	4.61 ± 1.45	4.08–5.14	0.778
≥9000	49 (61.25)	4.71 ± 1.62	4.25–5.18	
Duration of current stay (year)				
<10	40 (50.00)	4.57 ± 1.61	4.06–5.09	0.567
≥10	40 (50.00)	4.77 ± 1.49	4.29–5.25	
Living with smokers				
No	23 (28.75)	5.00 ± 1.68	4.27–5.73	0.236
Yes	57 (71.25)	4.54 ± 1.49	4.15–4.94	

^a^ Using one-way ANOVA testing *p*-value. * Statistically significant difference in mean BLLs across category (*p* < 0.05).

**Table 2:** The consumer goods of pregnant women (in the past month) and mean, 95% CI for BLLs (*n* = 80).

Consumer Goods	*n* (%)	BLLs (µg/dL)		*p*
		Mean ± SD	95% CI	
Coffee consumption				
No	74 (92.50)	4.59 ± 1.55	4.23–4.95	0.103
Yes	6 (7.50)	5.66 ± 1.21	4.39–6.93	
Milk consumption				
No	3 (3.75)	4.67 ± 0.58	3.23–6.10	0.992
Yes	77 (96.25)	4.67 ± 1.58	4.31–5.03	
Seafood consumption				
No	15 (18.75)	4.80 ± 1.97	3.70–5.89	0.731
Yes	65 (81.25)	4.64 ± 1.45	4.28–5.00	
Eyeliner use				
No	73 (91.25)	4.67 ± 1.55	4.31–5.03	0.945
Yes	7 (8.75)	4.71 ± 1.70	3.14–6.29	
Lipstick use				
No	29 (36.25)	4.90 ± 1.89	4.17–5.61	0.387
Yes	51 (63.75)	4.55 ± 1.32	4.18–4.92	
Brush on use				
No	54 (67.50)	4.76 ± 1.70	4.30–5.22	0.436
Yes	26 (32.50)	4.50 ± 1.20	4.01–4.99	
Nail varnish use				
No	72 (90.00)	4.62 ± 1.59	4.25–5.00	0.390
Yes	8 (10.00)	5.12 ± 1.12	4.18–6.07	
Household insecticide use				
No	70 (87.50)	4.63 ± 1.60	4.25–5.00	0.482
Yes	10 (12.50)	5.00 ± 1.15	4.17–5.83	

**Table 3:** The supplement intake of pregnant women (in the past month) and mean, 95% CI for BLLs (*n* = 80).

Supplement Intake	*n* (%)	BLLs (µg/dL)		*p*
		Mean ± SD	95% CI	
Iron				
No	4 (5.00)	4.50 ± 1.73	1.74–7.25	0.847
Yes	76 (95.00)	4.68 ± 1.55	4.33–5.04	
Calcium				
No	17 (21.25)	4.88 ± 1.45	4.13–5.63	0.537
Yes	63 (78.75)	4.62 ± 1.58	4.22–5.02	
Vitamin C				
No	66 (82.50)	4.60 ± 1.57	4.22–4.99	0.391
Yes	14 (17.50)	5.00 ± 1.41	4.18–5.81	

**Table 4:** Correlation coefficient (r) between BLLs and pregnant women’s health (*n* = 80).

Pregnant Women’s Health Factors	r	*p*
Gestational age (weeks)	−0.251	0.024 *
Pre-pregnancy BMI (kgm^−2^)	−0.124	0.272
Systolic blood pressure (mmHg)	0.271	0.015 *
Diastolic blood pressure (mmHg)	0.075	0.506
Hemoglobin (g/dL)	0.012	0.916

* *p* < 0.5 identify significant associations between BLLs and factors listed in the first column.

**Table 5:** Crude odds ratio (cOR) and adjusted odds ratio (aOR) for predictors of BLLs ≥ 5 µg/dL (*n* = 80).

Variable	cOR	95% CI	*p*	aOR ^a^	95% CI	*p*
Higher education level						
No	Ref			Ref		
Yes	0.20	0.04–0.97	0.045 *	0.16	0.03–0.80	0.027 *
Trimester						
First trimester	Ref			Ref		
Second trimester	2.18	0.73–6.53	0.164	2.23	0.69–7.18	0.177
Third trimester	1.81	0.38–3.65	0.773	1.40	0.42–4.68	0.584
Systolic blood pressure						
<120 mmHg	Ref			Ref		
≥120 mmHg	2.59	0.92–7.31	0.072	5.00	1.23–17.16	0.023 *
Diastolic blood pressure						
<80 mmHg	Ref			Ref		
≥80 mmHg	1.44	0.45–4.59	0.533	2.47	0.62–9.86	0.202
Hemoglobin level						
≥11 g/dL	Ref			Ref		
<11 g/dL	2.47	0.55–11.15	0.239	3.64	0.59–22.59	0.166

^a^ Adjusted for age, education, parity, gestational age, and pre-pregnancy BMI. * *p* < 0.05.

## Data Availability

Not applicable.
